# Theological Therapeutics

**Published:** 1856-08

**Authors:** 


// HALL'S JOURNAL OF HEALTH.

OUR LEGITIMATE SCOPE IS ALMOST BOUNDLESS: FOR WHATEVER BEGETS PLEASURABLE AND HARMLESS FEELINGS, PROMOTES HEALTH; AND WHATEVER INDUCES DISAGREEABLE SENSATIONS, ENGENDERS DISEASE.

VOL. III.] AUGUST, 1856. [NO. VIII.

### THEOLOGICAL THERAPEUTICS.

ABOUT a hundred and seventy years ago, there was a little boy; and he must have been a very little boy, for when he reached man's estate he measured scarcely five feet; and besides being, he was doing, to wit: he was on his knees in a school-room, while the schoolmaster in his gown was offering up the morning prayer, according to the good old Puritan custom. Our little hero's attention was attracted by sights rather than by sounds, and observing, between his fingers, (how many grown-up people do the same thing now-a-days on Sundays!) a little mouse crawling down the bell-rope, he was thoroughly aroused. Presently the mischievous little thing went up to the master, and began to tug away at the tail of his gown. The Dominie twitched, and the mouse twitched back again, but mousey had the advantage, for it had its eyes open, while it would have been greatly out of place for the Dominie to have opened his, although it was plain that he wanted to do it. It is needless to say, that the little scholar enjoyed the contest amazingly; and the sense of the ridiculous came over him so avalanchingly, that he could hold in no longer; he tried to, for it is very wicked to laugh at prayers, but the more he tried the more he couldn't, and out it came—the irrestrainable guffaw—full, loud, ringing. "Now, you little '*monkey*'"—the only epithet which the pious old teacher would allow himself—"if you don't make me a piece of poetry off hand, I'll punish you severely for such irreverence." The little culprit trembled, and well he might—who could write poetry under fear of the ferule? We have generally understood that poetry was the offspring of solicitation, not compulsion. Many poets solicit the Muses for an inspiration: not a few solicit brandy-and-water. But desperation nerves us to wonders, sometimes; and our little hero, more dead than alive, muttered out:—" *Please, Sir,*

> " A little rat, for want of stairs,
> Came down the rope to say his prayers."

It is needless to record the Dominie's mollification; and he, who afterwards became the almost (in our estimation) inspired Dr. WATTS, saved his skin whole at that time.

Now, to come to the point by way of persuasion; having roused your curiosity and your blood this hot day of midsummer, having no hope of doing it in any other way, we proceed to tell you, that this same renowned man must have been a kind of medical doctor as well as a theological, and an observant one, too, for he found out, what we have since done, and what we have repeated in our pages often, in consequence of its importance. We will give you his idea in our own words, for we think we have a knack of saying just exactly what we mean, and in such a way that it cannot mean anything else, and in such a way, too, that it does not give one the headache to study out its meaning. A great many "*inferences*" may be drawn from this subject.

*First:* an observer of mice in his youth, may become an observer of men in maturity. *Second:* The observation of little things makes the great man.

We want to have the influence of this great name in enforcing our views. We are well aware that there are multitudes of people who wouldn't care a fig what Dr. HALL says, but would be strongly influenced by any saying of Dr. WATTS, whether it pertained to theology or physic.

After all, we do not believe in the theory of the renowned Doctor. He observed a fact, which we receive as a fact, but the appendages thereto we do not allow. For example: he believed that the "*Adversary*," as FRIENDS call the Evil One, made use of high living to worry the Christian, and the more certainly to destroy the sinner. But for fear we may "put a point" on his discourse, which might not be allowed by others, we will give his language. " The Adversary is very busy at his mischievous work, especially when the powers of nature labor under any disease, and such as affects the head and the nerves; ever ready to fish in troubled waters, when the humors of the body are out of order."

Now, as dyspepsia makes "the powers of nature labor," and "affects the head and nerves," putting "the humors of the body out of order," and as high living, unphilosophical living, eating too much and exercising too little, originate these very things, it is a fair conclusion, without retracing each link of the chain, that religious enjoyment is diminished by sickness, and consequently health must promote it; and if health promotes religious enjoyment, while disease lessens it, the great fact forces itself on every reflecting mind, with a moral power which is irresistible—"HEALTH IS A DUTY."

Taking Dr. WATTS' theory that Satan uses ill-health as an instrument to "give greater disturbances to the mind, stimulating and urging to the unruly passions," then we do wrong to place that instrument in his hands, and we do right in studying how to keep that destructive weapon out of his hands; in other words, laying figures of speech aside, *we ought to study how to keep well, if we are so already; how to regain health, if we have lost it; as a means of enjoying that religion to the full whose end and aim is an immortality of bliss.*

